# Root patterning: it takes two to tangle

**DOI:** 10.1093/jxb/erw049

**Published:** 2016-02-19

**Authors:** Ben Scheres, Marta Laskowski

**Affiliations:** ^1^Plant Developmental Biology, Wageningen University Research, Building 107 (Radix), Droevendaalsesteeg 1, 6708 PB Wageningen, The Netherlands; ^2^Oberlin College, 119 Woodland, Oberlin, OH 44074, USA

**Keywords:** Arabidopsis, bending hypothesis, lateral root initiation, oscillation hypothesis, pattern formation, root architecture, waving growth

**The mechanisms that pattern lateral root primordia are essential for the elaboration of root system architecture, a trait of key importance for future crop breeding. But which are most important: periodic or local cues? In this issue of *Journal of Experimental Botany* (pages 1411–1420), Kircher and Schopfer manipulate growth direction to demonstrate the importance of both sources of patterning information.**

It may seem odd to use petri dishes to tease out the rules by which roots ramify underground – to aid the search for water and nutrients, circumnavigate soil particles and balance the forces on the aboveground plant for holdfast. But the simplicity of the ‘free running’ root-on-a-plate can reveal mechanisms of action that could easily go undetected when studying the system under more demanding conditions. Knowing how the basic mechanisms operate then helps us understand the underground plant system at a different level. This has led to precise descriptions of stages of lateral root primordium formation and outgrowth, the implication of many genes in this process, and to a recognition of the importance of overlying tissue in the emergence of lateral roots (see review by Peret *et al.*, 2009).

A particularly exciting question deals with lateral root patterning: where and when new lateral root primordia form. Together with the subsequent decision on how fast to grow out from these primordia, this process defines the basic features of root architecture. This question has been particularly puzzling because while there is some regularity to the spacing, the distance between individual lateral roots is not fixed. Indeed, it is far more variable than, say, the spacing of leaves about a stem.

## Parallel lines of evidence

**Fig. 1.**
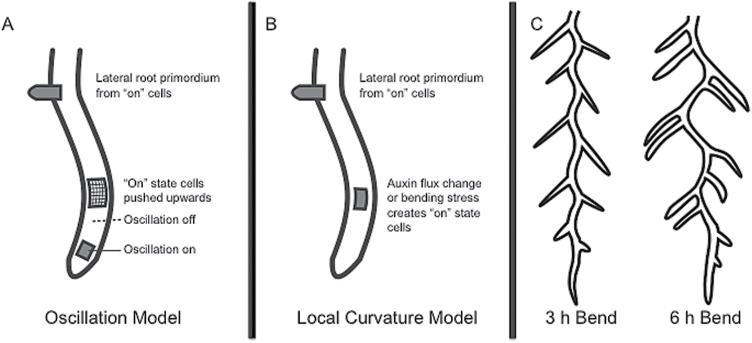
Combining local and global cues for lateral root patterning. (A) The oscillation model posits an oscillating gene program in the basal meristem; cells which have the program ‘on’ will carry this state with them during expansion and are competent to form primordia. (B) The local cue model posits that cell shape and auxin flux changes and/or bending stress signals lead to the activation of the lateral root program. (C) The constant frequency but altered left-right positioning of lateral roots at different induced bending intervals reveals both frequency and bending cues.

During the past decade two parallel lines of evidence have emerged from work on petri plates that emphasize either periodic or local cues for root primordium positioning (Laskowski, 2013; Van Norman *et al.*, 2013). So, on the one hand there is evidence that cyclic activation of an auxin response reporter in elongating cells at the root tip marks these as founder cells for lateral root primordia (Fig. 1A) (De Smet *et al.*, 2007; Moreno-Risueno *et al.*, 2010; Xuan *et al.*, 2015). On the other hand, it has been shown that local curvature of roots matters for the selection of sites of lateral root primordium formation (Fig. 1B) (Ditengou *et al.*, 2008; Dubrovsky *et al.*, 2008; Laskowski *et al.*, 2008; Richter *et al.*, 2009).

As frequently happens in science, particularly when interesting rival hypotheses emerge, both lines of evidence can be put diametrically against each other, holding that if either one of them turns out to be right, the other one must be wrong – a classic thesis–antithesis case. Cracks in that reasoning were already obvious from the published data. Neither theory for the origin of patterning information could easily explain all observations: local cues could not easily explain the correlation with oscillations (although the idea of spill-over from previous primordia was explored by Laskowski *et al.*, 2008); and global oscillations could not explain left-right patterning. Now Kircher and Schopfer (2016) provide the starting point for a synthesis of the existing ideas on patterning lateral root primordia. And the essentials of both ideas turn out to be correct.

## Elegant experiments

The elegance of Kircher and Schopfer’s work lies in the deconvolution allowed by the experimental setup. A classic lateral root primordium stain was shown to match well with transgenic reporter dynamics, and hence used to map how often primordia arise and how rapidly they mature under a variety of ‘petri dish’ environments. Careful measurements with optimized growth conditions showed that the rate of primordium formation and their average frequency did not vary much when adjustments to the orientation of the agar plates modulated the direction of root growth. This allowed the authors to uncouple the effect of local curvature (which determined whether lateral root primordia formed on the left or right side of the main root) from the frequency of induction.

The key data from their paper (see Fig. 1C) should suffice to convince aficionados of either curvature or frequency cues that neither one of them is enough to determine the pattern of outgrowth. The frequency at which lateral roots grow out remains almost constant even in situations where the frequency of curvature changes. At the same time, the position of the lateral roots is fixed to the convex side of the curve and tends to be focused towards its apex. At some frequencies of curvature, this results in more than one lateral root emerging on the convex part of a single curve, relatively near to the point of peak curvature (Fig. 1C). Hence, the position of lateral roots is determined by local curvature while the overall frequency is independent of the frequency of curvature.

Why was this seemingly simple observation not made before? A part of the explanation is coincidence. The authors present convincing evidence that the normal waving growth rhythm of roots on near-vertical petri dishes, which arises from a combination of gravitropic and thigmotropic responses, happens to give a curvature frequency that maps within the ‘one root per bend’ domain. However, there are other factors involved. A previous paper where bending frequencies were modulated reported differences in the frequency of lateral root formation (Lucas *et al.*, 2008). These same authors also reported arrested primordia in their growth conditions, which is not observed in the studies by Kircher and Schopfer (2016). Hence, conditions of growth can play a role in these assays, as can be expected when one considers the potentially elaborate controls on lateral root outgrowth in complex environments. Without calling any growth condition ‘better’ or ‘worse’, it seems fair to make the point that if one can stabilize one aspect of pattern formation (here, frequency) and make the other vary, the claim that both must be (able to be) controlled independently is valid.

## Synthesis and progress

The evidence presented here is an important step towards a synthesis. The proposal is that the variation in lateral root spacing may be explained by a combination of an oscillation of auxin response that establishes a region of competence, together with local cues including curvature that position primordia within that region. Curvature plays a role in such positioning, but other factors, such as hydropatterning (Bao *et al.*, 2014), also matter, and discovering how these factors are integrated remains a challenge. Communication between pre-existing lateral root primordia (Laskowski *et al.*, 2008) and events occurring in the oscillation zone may also explain part of the variation, subsequent to the first oscillation, and warrants further investigation.

The oscillation mechanism was discovered as a fluctuation in auxin response (De Smet *et al.*, 2007), and although not all auxin-responsive genes oscillate (Moreno-Risueno *et al.*, 2010), specific auxin sources in the root cap contribute to the oscillation (Xuan *et al.*, 2015). Subsequent to the oscillation, auxin and its transport are involved in many of the early steps of lateral root initiation that take place at the local site where a primordium is initiated on one or the other side of the axis (Benkova *et al.*, 2003; Okushima *et al.*, 2007; Dubrovsky *et al.*, 2008; De Rybel *et al.*, 2010). Even taking to heart the caveat that it is sometimes unwise to explain the unknown with the known, the re-occurrence of auxin in oscillatory priming and the initiation of lateral root primordium outgrowth means we have a good candidate for a synthesis of the two cues for lateral root patterning. After finding this out, the petri plates will have served their purpose and can be replaced by the underground challenges.
